# Personal barriers to participation in chosen instrumental activities of daily living among community-dwelling persons with schizophrenia in Rwanda

**DOI:** 10.4102/ajod.v12i0.1063

**Published:** 2023-06-28

**Authors:** Pierre D. Turikumana, Lizahn G. Cloete, Jerome P. Fredericks

**Affiliations:** 1 Department of Occupational Therapy, Faculty of Health Sciences, University of Rwanda, Kigali, Rwanda; 2 Department of Occupational Therapy, Faculty of Medicine and Health Sciences, Stellenbosch University, Cape Town, South Africa

**Keywords:** individual hindrances, schizophrenia, personal barriers, medication side effect isolation, instrumental activities of daily living, community participation, decreased motivation

## Abstract

**Background:**

Mental disorders are a major health concern across the globe. Schizophrenia, one of the mental disorders, affects approximately 20 million people globally and 5 million people within the African continent. Schizophrenia can affect all areas of life, including participation in instrumental activities of daily living (IADLs).

**Objectives:**

The study aimed to explore personal barriers affecting participation in chosen IADLs among community-dwelling persons with schizophrenia in Kigali city, Rwanda.

**Method:**

A qualitative, embedded case study design and constructivist epistemology paradigm were used. Purposive sampling and semi-structured interviews were conducted with 20 participants that included 10 persons diagnosed with schizophrenia (case 1) and 10 of their caregivers (case 2). Data were analysed according to the seven steps of Ziebland and Mcpherson.

**Findings:**

The two themes identified were community negative attitudes and individual hindrances to participation in IADLs. Theme 1 demonstrated the community’s poor support towards persons with schizophrenia due to the stigma attached to mental health illness, which is reported elsewhere. This paper reports on individual hindrances to participation, which revealed limited knowledge and skills, decreased motivation and interest, financial problems, maladaptive behaviours, medication side effects, loss of social interaction and isolation, and disorganised in performing activities to negatively affect persons with schizophrenia’s full participation in their chosen IADLs.

**Conclusion:**

Community-dwelling persons with schizophrenia are experiencing various hindrances to participating in their chosen IADLs, which shows a need for support from different stakeholders to improve access and participation of persons with schizophrenia in their daily activities based on their abilities.

**Contribution:**

Different barriers affecting participation of the persons with schizophrenia in their chosen IADLs were highlighted together with the common affected IADLs. It is recommended that when right support is provided, persons with schizophrenia may function at their maximum abilities in their activities of choice and may live at their highest independence level.

## Introduction

The World Health Organization stated that schizophrenia affects approximately 24 million people worldwide (WHO 2022). Schizophrenia is the most overwhelming condition among persons living with mental health conditions where persons experience disorganised behaviours and decreased ability for independence (Bifftu, Dachew & Tiruneh 2014). Schizophrenia is a serious psychiatric disorder affecting an individual’s thinking, emotions and behaviours. Persons with schizophrenia may experience hallucinations, delusions, disorganised behaviour or speech, negative symptoms and impaired cognitive functions (Rasool et al. 2018).

Considering a positive and negative syndrome scale (PANSS) classification of symptoms of schizophrenia, positive symptoms include sensory, behavioural and thought disturbances (Hellinger et al. 2019). Some of these include hallucinatory behaviours, delusions, thought disorganisation, ideas of persecution and aggression. Negative symptoms include reduced motivation, emotional withdrawal, apathy, stereotypical thinking and difficulties in abstract thinking (Hellinger et al. 2019). The negative symptoms experienced by persons with schizophrenia worsen the life skills such as self-awareness, problem-solving, decision making or coping with stress necessary for participation in instrumental activities of daily living (IADLs) and affect occupational functioning in general (Rabinowitz et al. 2012). Those IADLs are activities done by individuals every day to take care of themselves, their homes and their community such as shopping, caring for others, medication management, food preparation and others. Those life skills are the elementary skills that are required for living autonomously, improving meaningful life and fulfilling, fruitful roles (Lipskaya-Velikovsky, Jarus & Kotler 2017; Samuel, Thomas & Jacob 2018). Those positive symptoms can act as personal barriers to participation in community activities such as attending community meetings and participation in church activities.

Persons with schizophrenia experience difficulties with executive functioning, which affects functional independence and participation in chosen IADLs (Macedo et al. 2018; Ono et al. 2020), as well as difficulties in planning routines and tasks carried out daily. Functional IADLs greatly depend on cognitive aspects such as planning, organisation and memory (Contador et al. 2020). Through participation, people can gain knowledge and skills, organise time and convey structure to their lives. Consequently, a lack of opportunity or inability to participate in different activities can lead people to lose structure and daily organisation at home or in the community (James et al. 2019).

There is a Model Mental Health Care in Rwanda established in 2009 by Partners in Health (*Inshuti Mu Buzima*) in cooperation with the Rwanda Ministry of Health. That model aimed to provide mental health care at district hospitals through community education programmes supporting both service delivery and access to care (Partners In Health 2016). Moreover, few qualified mental health professionals are among the challenges to setting up a national mental health programme in Rwanda (Kayitashonga & Mohamand 2015), which might affect the barriers experienced by persons with schizophrenia in their daily participation.

Therefore, this article aimed to explore personal barriers affecting participation in chosen IADLs among community-dwelling persons with schizophrenia in Rwanda and to inform relevant stakeholders to address barriers encountered by persons with schizophrenia living in the community to improve their participation in IADLs. This article highlights caregivers’ perceptions and views towards personal barriers affecting participation in chosen IADLs among community-dwelling persons with schizophrenia in Kigali, Rwanda.

## Research method and design

A qualitative, embedded single case study design with a constructivist epistemology paradigm was used. A constructivist epistemology was most suited for this study as it allowed the researcher to interpret the participants’ views and perceptions as a way of generating knowledge about the personal barriers experienced by participants. Furthermore, a constructivism paradigm emphasises that humans construct their understanding and knowledge of the world through experiencing things and reflecting on those experiences (Adom, Attah & Ankrah 2016). Constructivists affirm that reality is subjective because it is from the participants’ perspectives (Adom et al. 2016).

A qualitative methodology enables the researcher to collect and analyse non-numerical data such as audio or text to understand concepts, opinions or experiences and analyses the phenomena in positions and meaning expressed by the individuals (Creswell 2009). An embedded single case study design uses multiple units of analysis and improves credibility through data source triangulation with a broad range of different participants’ perspectives (Lietz & Zayas 2010; Slack et al. 2015). Persons with schizophrenia were an integral part of the study where perceptions and views of caregivers were influenced with the barriers encountered by the persons with schizophrenia within their personal and social environment.

The persons with schizophrenia were defined as a case based on the following shared characteristics: having a diagnosis of schizophrenia, having a caregiver living in Kigali, scoring between 20 and 30 on the mini-mental status examination. Furthermore, a person with schizophrenia has to be a member of the OPROMAMER association and live in Kigali. On the other hand, the caregivers of persons with schizophrenia were defined as a case based on the following characteristics: being a caregiver of a person with schizophrenia and living in Kigali.

### Study setting

The study was carried out in Kigali, the capital city of Rwanda. According to the 2012 Population Census reported in 2014, Kigali has a population of 1 135 388 (Ndayisaba 2013). In Kigali, many people use public transport to travel to different working places, including where some IADLs are performed, such as the market for shopping, churches for religious and spiritual expression activities and hospitals for seeking health services and medication management.

### Study population, sampling strategy and selection criteria

Participants of this study were persons with schizophrenia and their caregivers living in Kigali. Qualitative researchers were advised by Aguboshim to adopt sample sizes that yielded saturation based on the expert researchers in the literature who have conducted similar studies in the past. Furthermore, it was recommended for qualitative researchers to use a minimum of 15 participants that may saturate between 20 and 30 interviews for the case study (Aguboshim 2021).

Purposive sampling was used for this study. This is a method of selecting participants based on the researchers’ judgement about the most informative and potential participants (Moser & Korstjens 2018). Twenty participants, including 10 persons with schizophrenia and 10 caregivers who participated in this study, were recruited to obtain in-depth information regarding the personal barriers affecting the participation of community-dwelling persons with schizophrenia in their chosen IADLs.

The following were specific selection criteria for the study participants. Persons with schizophrenia had to be 18 years old and above, live in Kigali city, be able to hear and communicate verbally, stable and have no comorbid physical conditions or intellectual disabilities. Persons with schizophrenia with the score between 20 and 30 on the Mini Mental State Examination (MMSE) (Attard 2017). Occupational therapist administered this test to each person with schizophrenia before interviewing the participants to test their mental state. Persons scored 20–24 were considered to have mild cognitive impairment, whereas those who scored 25–30 are cognitively able. The exclusion criteria were for those scoring below 20 because it indicates moderate to severe cognitive problems that could affect the interview process. Persons with schizophrenia should also indicate their consent to participate voluntarily in the study.

The caregivers of persons with schizophrenia had to be over 18 years old, have accepted the invitation to participate voluntarily in the study and be a primary carer involved in everyday care and/or providing support or assistance to an individual with schizophrenia participated in this study.

The recruitment of participants was achieved by requesting the contact details of participants from OPROMAMER association. This association strives to promote solidarity among people with mental disorders and works to transform the understanding of mental health in Rwanda. The author met the leader of the association, people with mental disorders and their caregivers to shortly discuss the research access to their information (mainly names, phone number and diagnosis for those people with mental disorders). This association organised meeting with persons with mental disorders, including schizophrenia, and sometimes involved their caregivers during the meeting and support group.

After receiving the phone contacts of the persons with schizophrenia and their caregivers, the author telephonically contacted the persons with schizophrenia for consent. Moreover, some persons with schizophrenia who did not have phones were telephonically contacted on the phones of their caregivers as the second option. Additionally, the caregiver-participants were contacted for consent telephonically after obtaining consent from the persons with schizophrenia to participate in the study.

The author and participants met at OPROMAMER association office, where the participants received the information leaflet translated into Kinyarwanda language as preferred by all the participants, and the author explained the information in the leaflet to the participants. The consent form translated into Kinyarwanda was also given and signed by the participants after deciding whether they wanted to participate in the study. For five persons who were unable to read, the author read the consent form and ensured that the participants understood what is in the consent form, and respected their rights to refuse or withdraw any time. Consent was also obtained for audio recordings of the interviews for later analysis by the author.

### Data collection

Semi-structured, one-on-one, face-to-face interviews were held at OPROMAMER association office. Interviews lasted between 20 and 30 min per interview. Most questions were open-ended both to persons with schizophrenia and their caregivers based on the interview guides to allow them to talk about personal barriers affecting participation in IADLs for community-dwelling persons with schizophrenia living in Kigali, Rwanda. The interview questions were asked to address the objectives of the study.

During the interview process, the persons with schizophrenia were asked to choose the activities they mostly do from the list of different IADLs. Those IADLs include care of others, care of pets, child-rearing, driving and community mobility, financial management, communication management, food preparation, shopping, religious and spiritual activities and expression. They also had to explain barriers encountered during those activities. After interviewing the persons with schizophrenia, the caregivers were interviewed one-on-one to explore in depth the personal barriers affecting participation in the chosen IADLs of the persons with schizophrenia. This interview method was used to maintain confidentiality and to create the opportunity for all participants to speak freely.

Paraphrasing and prompts were used as needed, especially for persons with schizophrenia, by rephrasing, repeating and summarising the questions to obtain clear responses. As the study was conducted in the period of the coronavirus disease 2019 (COVID-19) pandemic, all protective measures against this disease were considered including social distancing during the interviews, sanitising the hands before and after the interviews and wearing face masks.

### Data analysis

Data were analysed according to the seven steps suggested by Ziebland and Mcpherson (2006).

The transcription was the first step in data analysis where the interviews were transcribed from recorded interviews to text. The author and an experienced transcriber did the verbatim transcription. An experienced transcriber was explained prior by the author about the study and signed a confidential form saying that all information should be kept in private. The author read and re-read all transcripts while playing the audio recordings to correct any possible transcription errors. Each participant was assigned a code known only by the author to safeguard anonymity and confidentiality during the research process. The second step was reflecting on the data that were ensured during the data collection, whereby the author used a notebook and a pen to record the ideas contributing to the creation of codes and themes. The third step was coding, done through re-reading transcription and discussion with experienced supervisors during the coding process. Open coding was applied to each transcript to analyse the data.

The fourth step was actual analysis and was done in three steps. Step 1 entailed the grouping of codes, step 2 included the generation of categories and during step 3, themes were formed for persons with schizophrenia (case 1). The analysis of caregiver (case 2) information was also done in similar ways to the persons with schizophrenia in three steps. The cross-case analysis was done by comparing the findings from the persons with schizophrenia and their caregivers where similarities and differences were explored. Step five sought analytic depth and was achieved by incorporating the new insights and interpretations from the study supervisors related to the study.

The sixth step was testing and confirming the results were attained through data triangulation by collecting the data from multiple data sources, including persons with schizophrenia, their caregivers and the recent literature review to support the findings. The writing up was the last step where the author made sure that the findings reflected the experiences expressed by the research participants.

### Ethical considerations

This study was granted an ethical approval by the Health Research Ethics Committee at Stellenbosch University (Ethics reference number: S20/11/338) and by the Institutional Review Board at the University of Rwanda, College of Medicine and Health Sciences (Approval notice: No 068/ CMHS IRB/2021) as the requirement to conduct a study in Rwandan community. The ethical principles ensured during the study were: beneficence, non-maleficence, autonomy, confidentiality and justice (Bell & Bryman 2007). According to the Declaration of Helsinki, the study followed the ethical principles for medical research involving human subjects (WMA 2013).

To ensure beneficence, participants were informed that there was no direct benefit from their participation in the study; however, all research participants were given reimbursement for lunch and transport. The persons with schizophrenia and their caregivers were informed that they might benefit from this study by the time experienced different stakeholders in charge will address barriers. Non-maleficence and autonomy, such as not to harm the participants, were considered during the research process. It was planned that if the participants show emotional or psychological distress during the interviews, the interview question might be changed or the interview might be ended. The participants were also informed that they were free to withdraw from the study at any time. Referral for counselling was planned in case of severe emotional or psychological distress by having a clinical psychologist in place. To ensure the confidentiality of the participants, the author assigned a code (pseudonym) for each participant during the data transcription process and ensured at all times that the information were stored where external parties could not see it. The assigned codes can be understood as follows: (1) P indicates a person with schizophrenia; (2) C indicates a caregiver, (3) the number, like 1, 2 up until 10, indicate the place at which the participant interviewed and (4) G or K indicates the district in which the participant is located. The letter G indicates the Gasabo district while the letter K indicates the Kicukiro district. Furthermore, to uphold justice, the study participants were voluntarily requested to give informed consent to participate in the study. The study does not exclude any participants based on their educational level, socio-economic status or gender.

### Rigour

The author ensured that the study obtained accurate information by ensuring rigour, including credibility, transferability, dependability and confirmability. Strategies to ensure credibility, such as peer debriefing, were achieved through working with a qualified occupational therapist who reviewed the transcripts to ensure the accurateness of the data from the participants. Study supervisors also provided feedback on data analysis and the research process to avoid the author’s prejudices. Transferability ensured through the thick description by providing the details of the participants, their settings and the research methodology used to help the reader determines the transferability to different settings. Dependability was achieved through an audit trail where the author kept the record of the raw data and personal notes; a thick description detailing the description of research mainly data collection and analysis process was provided. Furthermore, peer review through the regular discussions and meetings with the study supervisors was convened where the feedback from these meetings was considered during the study. Confirmability was ensured by keeping the record of all raw data, such as the audio-taped interview, written field notes and verbatim transcription of the interviews. Data triangulation was ensured by collecting data from multiple data sources, including persons with schizophrenia, their caregivers and the recent literature review to support the findings.

## Findings

### Participant demographic data

The ages of participants from case 1 ranged between 29 and 76 years, which consisted of six women and four men. Eight participants resided in the Gasabo district, whereas two resided in the Kicukiro district during data collection. Two participants from this case scored between 20 and 24, and eight scored 25–30 on the Mini-Mental Status Examination. The scores of 20–24 indicate that the participants have a mild cognitive impairment and may require some supervision, support and assistance in their day-to-day functioning. The scores of 25–30 indicate that participants have no cognitive impairment; however, the participants might experience some deficits in day-to-day functioning especially in the most difficult activities of daily living. During the data collection process, the author occasionally repeated the questions and provided enough time to facilitate the participants to understand the questions, have enough time to think and provide the responses.

The ages of participants for case 2 ranged between 25 and 70 years. The caregivers were mostly the spouses of the persons with schizophrenia, the siblings of the persons with schizophrenia or the parents of the persons with schizophrenia. Eight women and two men participated in the study. Eight caregivers lived in the Gasabo district while two lived in the Kicukiro district during data collection.

Following within-case analysis, 17 sub-categories were formed for case 1 (persons with schizophrenia) and 20 sub-categories for case 2 (caregivers). Seven categories were generated based on similar sub-categories for case 1 and eight categories for case 2. One theme was generated for case 1 and one theme for case 2 through merging similar categories (Table 1). Furthermore, cross-case analysis was done by exploring similarities and differences, making links and identifying trends between the findings from the persons with schizophrenia and their caregivers.

**TABLE 1:** Themes, categories and sub-categories.

Themes	Categories	Sub-categories
**CASE 1 (Views from persons with schizophrenia)**		
1. Individual hindrances to participation	1.1 Limited knowledge and skills	1.1.1 Inability to use materials 1.1.2 Difficulty in remembering
	1.2 Decreased motivation and interest	1.2.1 Low confidence 1.2.2 Decreased motivation
	1.3 Financial problems	1.3.1 Lack of financial support 1.3.2 Lack of access to bank loans
	1.4 Maladaptive behaviours	1.4.1 Physical fights 1.4.2 Disorientation
	1.5 Medication side effects	1.5.1 Decreased body strength and energy
	1.6 Loss of social interaction and isolation	1.6.1 Avoiding contact with others 1.6.2 Limited social interaction
	1.7 Affected IADLs	1.7.1 Child rearing 1.7.2 Financial management 1.7.3 Medication management 1.7.4 Food preparation 1.7.5 Household activities 1.7.6 Religious and spiritual activities and expression
**CASE 2 (Perspectives from caregivers)**		
1. Individual hindrances to participation	1.1 Limited knowledge and skills	1.1.1 Poor planning 1.1.2 Difficulty in remembering
	1.2 Decreased motivation and interest	1.2.1 Decreased motivation 1.2.3 Loss of interest
	1.3 Financial problems	1.3.1 Lack of money 1.3.2 Poverty in the family
	1.4 Maladaptive behaviours	1.4.1 Aggression 1.4.2 Wandering
	1.5 Medication side effects	1.5.1 Body weakness 1.5.2 Sleeping too much
	1.6 Loss of social interaction and isolation	1.6.1 Not talking to others 1.6.2 Isolation
	1.7 Disorganised in performing activities	1.7.1 Not completing activity 1.7.2 Lack of control
	1.8 Affected IADLs	1.8.1 Care of pets 1.8.2 Financial management 1.8.3 Medication management 1.8.4 Household activities 1.8.5 Food preparation 1.8.6 Religious and spiritual activities and expression

*Source:* American Occupational Therapy Association, 2020, ‘Occupational Therapy Practice Framework: Domain and Process (4th ed.)’, *American Journal of Occupational Therapy* 74(2), 1–87. https://doi.org/10.5014/ajot.2020.74S2001

IADL, instrumental activities of daily living.

### Case 1: Theme 1: Individual hindrances to participation

This theme has seven corresponding categories. An individual hindrance in this study explains anything, actions or behaviours experienced by persons with schizophrenia that negatively affect their participation in chosen IADLs.

#### Category 1: Limited knowledge and skills

The findings of this article on individual hindrances to participation reported by the persons with schizophrenia showed limited knowledge and skills and memory problems to affect their participation in chosen IADLs like phone use and medication management.

Some IADLs performed by persons with schizophrenia were negatively affected by the inability and lack of familiarity with using some materials utilised to perform IADLs like phone.

‘I don’t use phone so much… because of not knowing it.’ (P1G)

‘Yeah, an individual gains knowledge and skills day by day. Therefore, sometimes I find myself having limited knowledge and confidence depending on the activity or daily circumstances.’ (P5G)

‘… I may forget. But that time, they remind me to take medications and I used water to swallow it.’ (P4G)

#### Category 2: Decreased motivation and interest

The study findings showed that persons with schizophrenia faced decreased volition and interest in performing their chosen IADLs. Some persons with schizophrenia experienced decreased self-confidence and motivation levels and stopped their chosen IADLs when they failed to accomplish them.

‘I feel bad. And I don’t continue to do different activities.’ (P3G)

‘My ability is low so that I cannot fulfill activities I want to do, then I immediately feel awkward, lose hope, like I cannot do anything.’ (P7K)

‘Sometimes my motivation is low that I am unable to care for my goats.’ (P8G)

#### Category 3: Financial problems

Many participants with schizophrenia reported financial problems as a major barrier that impacted their participation in their chosen IADLs. Some participants with schizophrenia reported a lack of financial support for their most liked IADLs, such as medication management, and financial management.

‘I am a talented musician. I know how to compose and sing, so if I get financial support to produce my songs this will help me. Here I want to say having a studio which will record my songs. This will be my way of delivering my message to all persons with mental health illnesses and others in the community.’ (P9G)

‘The challenge I had is taking medication because the medication is too expensive.’ (P10G)

‘What I need much is financial support. I have so many projects, but I can’t afford to realise them. I have an idea of keeping chickens and others, but I don’t have excessive desire. We have a group where we can borrow money and bring it back in 3 months. We cannot access loans from banks because when you have the history of being in CARAES (neuropsychiatric hospital), they can’t risk giving you money; they think you can misuse it.’ (P2G)

#### Category 4: Maladaptive behaviours

The findings of this study revealed that most of the participants exhibited maladaptive behaviours such as physical fights and disorientation when they relapse that hindered their participation in their chosen IADLs. Persons with schizophrenia reported that their symptoms negatively influence their participation in their chosen IADLs.

‘… I feel agitated sometimes. There is a time where I fight against my mother. This affected my participation in independent living activities because I spent a time at the hospital doing nothing.’ (P6G)

‘… I feel disoriented. These symptoms affect my participation in independent living activities because I spent time at the hospital doing nothing.’ (P6G)

#### Category 5: Medication side effects

The findings of this study showed that most of the participants with schizophrenia reported that medication side effects such as diminished body strength and extreme tiredness hinder their participation in their chosen IADLs.

‘Activities that required me to spend a lot of time walking, those activities are tiresome for me, because of medication side effects as I have been using Haldol and my neck gets stiffness.’ (P6G)

#### Category 6: Loss of social interaction and isolation

The study’s findings revealed that almost all participants with schizophrenia reported limited social interaction and isolation as barriers hindering their participation in chosen IADLs.

‘Most of the time I do them alone and this caused me to not finish this activity. I become bored.’ (P7G)

‘Oooh!! No, I go on my own without looking or talking to others.’ (P9G)

#### Category 7: Affected instrumental activities of daily living

The most affected IADLs reported by persons with schizophrenia include medication management reported by five participants, household activities reported by four participants, food preparation and religious and spiritual activities reported by three participants.

‘I may forget. But that time, they remind me to take medications and I used water to swallow it.’ (P4G)

### Case 2: Theme 1: Individual hindrances to participation

This theme emerged from the caregivers and is composed of eight categories.

#### Category 1: Limited knowledge and skills

More than a half of the caregivers reported that the persons with schizophrenia presented cognitive problems including poor planning and difficulty remembering when participating in their chosen IADLs and reported that some of them require reminders:

‘Most of the time she doesn’t know how to organize, to start, and to complete the activities when she is alone at home.’ (C7G)

‘… When no one is at home, he does nothing, and he doesn’t care his hair look and animals are not cared for. Before he could try, but now he doesn’t even understand, even when you tell him something he keeps quiet. It requires reminding him many times, he is no longer able to cook, and remembering medications is hard … we always remind him.’ (C8G)

#### Category 2: Decreased motivation and interest

Some caregiver participants reported decreased motivation and interest to negatively impact the participation of the persons with schizophrenia in their chosen IADLs:

‘He is not motivated at all. The only thing he tries to do, is keeping goats, but no motivation.’ (C8G)

‘… [*H*]e likes being calm and does nothing, however, when someone reminds him, he (person with schizophrenia) does the required activity after being explained that something is so important for the benefit of his life.’ (C1G)

#### Category 3: Financial problem

The financial problem was reported by many caregivers to limit the participation of the persons with schizophrenia in their chosen IADLs, especially care of others, pets, medication management and shopping:

‘… [*I*]n case she has financial problems, to care about her activities becomes difficult.’ (C2G)

‘… When patients go back in the family which is poor where even eating is a problem, it is a big problem. Some of the patient may even leave medications because of that problem.’ (C9K)

‘When she is healthy, she tells me ideas of what we can do, like trading tomatoes. But because there is no financial ability, you can’t know that she can do this or that. She might be having the ability to do things but, no money.’ (C5G)

#### Category 4: Maladaptive behaviours

Many caregivers reported some maladaptive behaviours demonstrated by the persons with schizophrenia, such as aggression and wandering mainly during the crisis condition and have negative impacts on the participation of persons with schizophrenia in their chosen IADLs:

‘Signs are laughing and talking, and sometimes she tells me that I am the one who is sick. She even brings medication and tells me to take them as I am the one who’s sick. She wakes in middle of the night and takes a bath … when she is in crisis, she can even bring water from the spring, and she pours it all on her body.’ (C5G)

‘He doesn’t work, he only lay down. One day, he was attacked by crisis at work, he left the working place and was directing the cars in the street.’ (C10K)

#### Category 5: Medication side effects

Many caregivers testified that side effects of the medications taken by the persons with schizophrenia have a negative impact on their participation in the chosen IADLs. The reported medication’s side effects include body weakness, tiredness and sleeping too much:

‘He is not able, because it’s been a long time that he has this disease and medications that he takes everyday have induced body weakness overtime. It’s obvious that he is weak.’ (C1G)

‘… [*M*]edications that these patients take, weaken their bodies so much. When patients go back in the family which are poor where even eating is a problem, it is a big problem. Some of the patients may even leave medications because of that problem.’ (C9K)

‘… She told me that she was tired and every time she was sleeping.’ (C6G)

#### Category 6: Loss of social interaction and isolation

Most of the caregiver participants stated that persons with schizophrenia find it difficult to socially interact with others, thereby hindering their participation in their chosen IADLs. The caregiver participants noticed that persons with schizophrenia experience problems keeping the conversation going which impacts their participation in IADLs requiring verbal expression at home and in the community. Also, the caregivers explained that persons with schizophrenia face self-isolation, which impacts their participation in chosen IADLs that must be carried out in groups of many people.

‘Sometimes he is so quiet, and you see that he doesn’t want to talk to others.’ (C1G)

‘When you tell him something he keeps quiet.’ (C8G)

‘Most times she is alone.’ (C7G)

#### Category 7: Disorganised in performing activities

Many caregiver participants reported disorganisation such as not completing activity started and a lack of control among the persons with schizophrenia when participating in their chosen IADLs:

‘She loves working and shows that she wants it, but she just can’t. Before, she could peel potatoes for us, but now she can’t. She can even take the charcoal to her bed and grinds it and when you ask her what she is doing she says, well, I am cooking. So, all that you said she really wants it, but she just can’t…’ (C3G)

‘What I can say is that his disease makes him lose control.’ (C1G)

#### Category 8: Affected instrumental activities of daily living

Six IADLs were reported by the caregivers to be affected due to individual hindrances. Four of them were mostly reported, including medication management, food preparation and religious and spiritual activities, each activity reported by four caregivers, while care of pets was reported by three caregivers:

‘[*H*]e is becoming weaker. Previously, he [*person with schizophrenia*] could keep domestic animals and he did it perfectly, but now he is not being cared at home and he needs someone to remind him many times. He is no longer able to cook, and remembering medications is hard.’ (C8G)

### Cross case analysis from case 1 and case 2

Individual hindrances were reported by both persons with schizophrenia and their caregivers to negatively affect the participation of persons with schizophrenia in their chosen IADLs. Half of the persons with schizophrenia reported limited knowledge and skills in using some materials, including phones, and difficulties in remembering to take medications as hindrances to participating in medication management. In contrast, a greater number of their caregivers reported limited knowledge and skills in planning, which affected the financial management activity among persons with schizophrenia.

Persons with schizophrenia showed financial problems, including a lack of access to loans that affect their participation in caring for others, buying medication and caring for domestic animals like keeping chickens. However, most of their caregivers reported that the main source of financial problems is poor planning and abnormal behaviour because persons with schizophrenia spend money everywhere while trading. Most caregivers reported body weakness and sleeping too much due to medication side effects as an individual hindrance to participating in household activities. In contrast, many persons with schizophrenia reported decreased body strength in food preparation and participating in activities requiring walking a long distance to work.

Both persons with schizophrenia and their caregivers reported five commonly affected IADLs due to individual hindrances: financial management, medication management, food preparation, household activities, religious and spiritual activities and expression. However, only caregivers reported care of pets, whereas only persons with schizophrenia reported child rearing. This study shows that participation in a single IADL (like domestic activities) might be affected by more than one barrier, like limited knowledge and skills and body weakness due to medication side effects.

Therefore, based on the study findings, most of the barriers reported by persons with schizophrenia are in line with the barriers reported by their caregivers, even though there are other barriers either reported by persons with schizophrenia alone or by caregivers. An example of similarities in response for both participants includes medication side effects, where the person with schizophrenia stated, ‘When I took medications, they had strong effects on me’ (P2G) and the caregiver reported that:

‘She [*person with schizophrenia*] doesn’t do her activities when she immediately takes medications, because the medications have strong side effects on her. She immediately goes to bed after taking those medications.’ (C4G)

## Discussion and conclusions

This paper focused on exploring the personal barriers affecting participation in chosen IADLs among community-dwelling persons with schizophrenia living in Kigali, Rwanda. The study revealed that persons with schizophrenia reported limited knowledge and skills, including their ability to access information and memory problems that hinder their participation in chosen IADLs such as taking medication, meal preparation and phone use. On the other hand, most of the caregivers also reported limited knowledge and skills of the persons with schizophrenia, which includes forgetting to take medications, limited knowledge in financial management, a lack of concentration and poor planning of daily activities such as the time of taking medication, caring of pets and the time of cooking. Similarly, Macedo et al. (2018) conducted a qualitative study where caregivers reported that persons with schizophrenia experienced difficulties in planning routines and tasks. Cognitive dysfunction in schizophrenia is linked to poor social functioning, which impacts the accomplishment of the activities requiring higher executive functioning including IADLs and affects other functional outcomes (Contador et al. 2020; Shimada et al. 2016). The decreased ability to function independently in the home environment was reported by the family members of the persons with schizophrenia (Krupchanka et al. 2018).

This paper found that self-isolation, decreased motivation and limited social interaction hinder the engagement and participation of persons with schizophrenia in IADLs such as household activities. The loss of interest in those activities also hinders the participation of their family members who live with the persons with schizophrenia. This finding is supported by Macedo et al. (2018), who reported decreased volition among persons with schizophrenia. The persons with schizophrenia experienced self-isolation, low self-confidence and limited social interaction, as reported by caregivers. Those barriers were found to contribute to poor performance in household activities. Bhuyan, Chaudhury and Saikia (2016) found that social embarrassing behaviours had a negative impact on participation in IADLs. Horsselenberg et al. (2016) showed that victimisation and symptoms led to self-stigma and contribute to poor self-esteem among persons with schizophrenia. Therefore, there is a need to boost self-esteem, self-confidence and motivation of persons with schizophrenia to improve their participation in their daily activities and social interaction with other community members.

The maladaptive behaviours were found to hinder the participation of persons with schizophrenia in chosen IADLs. When these behaviours are expressed, persons with schizophrenia are taken to the hospital and lose their daily functioning at home or in their community. Ayres and Panickacheril (2015) found that abnormal motor behaviours created difficulties in goal-directed behaviours, which limited the accomplishment of different daily activities and affected the competencies of persons with schizophrenia to live and participate independently in the community. Bhuyan et al. (2016) revealed that socially embarrassing behaviour is high among persons with schizophrenia, and these behaviours negatively impact their participation in IADLs demanding social interaction and routines. Therefore, it is paramount that the family and community members helping persons with schizophrenia realise the need for early support and treatment.

This study found that medication side effects hinder the participation of persons with schizophrenia in medication management, household activities, religious and spiritual activities and food preparation. The medication’s side effects were stated to reduce physical strength, cause too much sleeping and cause extreme tiredness when participating in different IADLs. Some persons with schizophrenia stop taking those medications as a result of their side effects. These findings are supported by the literature, where mental health service users reported the problem of treatment availability due to the side effect of the medication (Dockery et al. 2015). The medication side effects were also reported by both persons with schizophrenia and caregivers to affect the health of persons with schizophrenia (James et al. 2019).

Therefore, mental health professionals should be aware that the side effects of medications taken by persons with schizophrenia affect their participation in their daily activities. Through the provision of available medication with minimal side effects, clear explanations of the side effect of medication taken, and management of these side effects, establishing good community follow-up of the clients would be beneficial to reduce the medication side effects and the number of hospitalisations.

Both persons with schizophrenia and caregivers reported that financial problems affect the participation of persons with schizophrenia in IADLs. The lack of financial support from the family and government and the inability to access bank loans limited their participation in chosen IADLs, such as caring for children, shopping, purchasing and managing medications and attending medical appointment at the health facilities due to poverty. Mental health service users and their caregivers reported difficulties affording financial costs, problems with transport facilities, inadequate governmental and social services and a lack of needed support for persons with schizophrenia (James et al. 2019; Krupchanka et al. 2018). Therefore, support to the socio-economically disadvantaged families with persons with schizophrenia may help them to improve their participation in their needed and chosen activities such as medication management, shopping and other activities of their choice. Providing health insurance covering the cost to disadvantaged persons with schizophrenia would help them more easily access health services. The decentralisation of mental health services provision at the community level may also help the mental health service users to have easier access to treatment.

The above findings are linked to the results from the study done by Samuel et al. (2018), where the impacted IADL activities were medication management (86%), food preparation (85%), shopping activities (78%), financial management (61%) and housework activities (47%) among the persons with schizophrenia. It was also stated that persons with schizophrenia still face problems participating in financial management activities (Dockery et al. 2015). Occupational therapists put more emphasis on the participation of individuals in occupations or activities. Therefore, they should be aware that persons with schizophrenia experience different personal barriers, such as cognitive problems, disorganisation in IADLs and decreased motivation. These barriers should be addressed before they are discharged to ensure maximum participation in social community activities. Regardless of these barriers, when support from relevant stakeholders is provided, individuals with schizophrenia can participate in their chosen activities and live productively in their families and communities.

### Limitations

This study reported a few limitations. The study participants were all involved in activities of the OPROMAMER association for the members who live in Kigali city, which might impact the barriers they face compared with those not members of the association. Due to the study design, it is difficult to generalise the findings. However, the concept of thick description was ensured through a detailed description of the participants, their setting and the research methodology used to help the reader to determine the transferability to a different setting. Also, purposive sampling was used in the selection of the participants based on the selection criteria. Another limitation of the study is the use of single method of data collection which is interview. However, the researcher collected data from both persons with schizophrenia and their caregivers which have contributed in having varieties of data.

### Recommendations

This paper recommends future studies to explore how cognitive problems encountered by persons with schizophrenia affect their participation in IADLs and the strategies to overcome personal barriers affecting the participation of persons with schizophrenia in their chosen IADLs activities. Further research might be conducted in the rural area for non-OPROMAMER association members to explore barriers affecting the participation of persons with schizophrenia in activities of their choice.
